# Brain-Computer interface control of stepping from invasive electrocorticography upper-limb motor imagery in a patient with quadriplegia

**DOI:** 10.3389/fnhum.2022.1077416

**Published:** 2023-01-09

**Authors:** Iahn Cajigas, Kevin C. Davis, Noeline W. Prins, Sebastian Gallo, Jasim A. Naeem, Letitia Fisher, Michael E. Ivan, Abhishek Prasad, Jonathan R. Jagid

**Affiliations:** ^1^Department of Neurological Surgery, University of Pennsylvania, Philadelphia, PA, United States; ^2^Department of Biomedical Engineering, University of Miami, Miami, FL, United States; ^3^Department of Electrical and Information Engineering, University of Ruhana, Hapugala, Sri Lanka; ^4^Department of Neurological Surgery, University of Miami, Miami, FL, United States; ^5^Miami Project to Cure Paralysis, University of Miami, Miami, FL, United States

**Keywords:** brain-computer interface, electrocorticography, spinal cord injury, gait, lower-extremity

## Abstract

**Introduction:** Most spinal cord injuries (SCI) result in lower extremities paralysis, thus diminishing ambulation. Using brain-computer interfaces (BCI), patients may regain leg control using neural signals that actuate assistive devices. Here, we present a case of a subject with cervical SCI with an implanted electrocorticography (ECoG) device and determined whether the system is capable of motor-imagery-initiated walking in an assistive ambulator.

**Methods:** A 24-year-old male subject with cervical SCI (C5 ASIA A) was implanted before the study with an ECoG sensing device over the sensorimotor hand region of the brain. The subject used motor-imagery (MI) to train decoders to classify sensorimotor rhythms. Fifteen sessions of closed-loop trials followed in which the subject ambulated for one hour on a robotic-assisted weight-supported treadmill one to three times per week. We evaluated the stability of the best-performing decoder over time to initiate walking on the treadmill by decoding upper-limb (UL) MI.

**Results:** An online bagged trees classifier performed best with an accuracy of 84.15% averaged across 9 weeks. Decoder accuracy remained stable following throughout closed-loop data collection.

**Discussion:** These results demonstrate that decoding UL MI is a feasible control signal for use in lower-limb motor control. Invasive BCI systems designed for upper-extremity motor control can be extended for controlling systems beyond upper extremity control alone. Importantly, the decoders used were able to use the invasive signal over several weeks to accurately classify MI from the invasive signal. More work is needed to determine the long-term consequence between UL MI and the resulting lower-limb control.

## 1 Introduction

Brain-computer interfaces (BCI) are a burgeoning technology promising improved quality of life for individuals affected by a broad range of neurological diseases or injuries (Robinson et al., 2021). BCIs strive to achieve functional improvements by circumventing the damaged nervous system to enable control of external devices and systems. This control has been demonstrated in brain-driven motor control, ranging from control of exoskeletons (Benabid et al., 2019) or muscle reanimation (Ajiboye et al., 2017) to control of motor intent-to-text systems for improved communication (Willett et al., 2021). In this capacity, BCIs seek to restore lost function as assistive devices.

Many BCI systems have been explored as tools in both able-bodied and motor-impaired subjects including those with amyotrophic lateral sclerosis (Vansteensel et al., 2016; Speier et al., 2017), stroke (Ang et al., 2015; Biasiucci et al., 2018), and spinal cord injury (SCI; Bouton et al., 2016), among others. With the need to address improved upper limb (UL) control (Anderson, 2004), many BCI endeavors have used neural signals to recreate reaching and grasp control (Nakayashiki et al., 2014; Bouton et al., 2016; Ajiboye et al., 2017; Benabid et al., 2019; Cajigas et al., 2021; Mencel et al., 2021). However, lower-limb (LL) motor impairment is often concomitant in these pathologies, and in the case of SCI, essentially all cases of damage to the spinal cord result in decreased function in the lower extremities. Though studies have used BCI control signals for LL experiments (King et al., 2013; Zhang et al., 2015; Qi et al., 2021; Zhao et al., 2022), many developments in rehabilitation and assistive devices focus primarily on UL control (Robinson et al., 2021). Additionally, most LL BCI systems have used non-invasive recording methods (Camargo-Vargas et al., 2021) at the expense of more reliable brain data with better spatial resolution (Robinson et al., 2021). Exploring invasive methods for how neural control signals extracted from the brain relate to LL movement can inform assistive device development and rehabilitation protocols that target multiple disorders affecting gait and volitional movement.

BCI applications for either assisting movement (He et al., 2018) or engaging in LL rehabilitation (Camargo-Vargas et al., 2021; Robinson et al., 2021) are varied in their approaches to extracting, decoding, and analyzing signals from the brain and are varied in the devices and systems these signals control. The use of invasive signals to drive LL robotics and orthotics is relatively new (Benabid et al., 2019), as prior to 2018 essentially all LL studies in humans have utilized EEG signals (He et al., 2018). The set of LL instructions most often presented to subjects includes motor imagery commands to distinguish between standing and walking (He et al., 2018), turning right or left, and using imagined UL movements to drive wheelchair control (Huang et al., 2012). Decoding architectures for these LL instructions have included frequency-domain analysis (Huang et al., 2012), machine learning (Jiang et al., 2015; Liu et al., 2017; Vouga et al., 2017), and more recently deep-learning techniques (Tortora et al., 2020; Hamid et al., 2022), yet many of these decoding studies are performed in healthy human subjects rather than their target subject populations who would benefit most from LL assistive systems.

The role of BCI-driven assistive devices and rehabilitative function is evolving. UL rehabilitation using BCI and functional electrical stimulation (FES) following stroke is among the most effective BCI rehabilitation paradigms demonstrated (Khan et al., 2020) and also shows promise for LL rehabilitation (Chung et al., 2015). LL BCI-FES rehabilitation for SCI recently demonstrated promising feasibility (Shokur et al., 2018), and though there are potential rehabilitation benefits using exoskeletons (Donati et al., 2016) as well, more work in target populations is required (Robinson et al., 2021).

Working with a subject, previously equipped with a fully implanted neural interface system (Cajigas et al., 2021), we tested the hypothesis that our subject could employ UL motor imagery to initiate stepping motion on a weight-supported gait rehabilitation treadmill better than inefficient performance defined as 70% (Kübler et al., 2001).

## 2 Methods

### 2.1 Participant

The research participant in this study was a 22-year-old right-handed male subject who was diagnosed with cervical spinal cord injury (ASIA C5) due to a motor vehicle accident 5 years prior. The research subject’s participation in this study was part of a clinical trial (ClinicalTrials.gov: NCT02564419) in which they were enrolled.

### 2.2 Neural data acquisition

**Figure 1:**
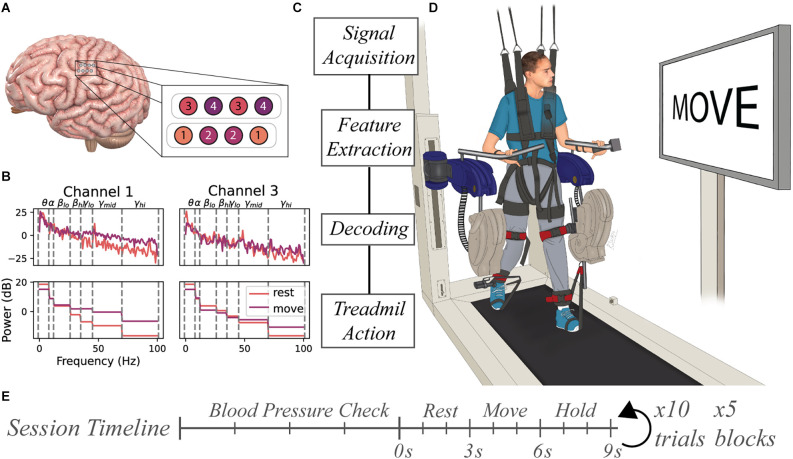
Experimental setup for BCI-controlled ReoAmbulator. **(A)** Two 4-contact electrode strips were implanted over the hand region of the sensorimotor cortex. The electrodes were configured in a bipolar mode resulting in four channels of ECoG data. **(B)** Features were extracted by computing the mean spectral power of select frequency bins for each trial. These features were used for decoder training and testing. **(C)** General flow diagram of information processing. **(D)** Motor imagery instructions were presented to the subject using a large display associated with the ReoAmbulator. Signals collected during instruction presentation would be collected, processed, and decoded. Correctly decoded signals triggered four to six gait cycles of walking on the ReoAmbulator system. **(E)** Session timeline overview. Each session began with three to four blood pressure measurements to assess orthostatic hypotension followed by the experimental procedure with repeated blocks of 10 trials. Each trial consisted of approximately 3 s of rest, 3 s of movement, followed by a hold period for treadmill stepping.

Neural data was collected from the research participant using two four-contact strip electrodes (Resume II Leads, Medtronic, Minneapolis, MN) that were surgically implanted under the dura in the hand knob region of the sensorimotor cortex approximately 4 months prior to ambulatory testing. The leads were subcutaneously connected to a stimulus pulse generator (Activa PC+S, Medtronic, Minneapolis, MN) capable of recording electrocorticography (ECoG) potentials from the lead contacts. The eight contacts were configured in bipolar mode resulting in four channels of ECoG data (Figure 1A). Internally, the device performed a 0.5–100 Hz band pass pre-amplifier filter on recorded ECoG potentials for all channels. The device allowed for the four channels to be configured as two time-series channels (channels 1 and 3) sampled data at 200 Hz while the other two were power channels (channels 2 and 4) that sampled the average power spectra of the signal between 4 Hz and 36 Hz every 5 s.

The implanted pulse generator internally buffered the data from all four channels at 400 ms intervals. These buffered data were transmitted in packets and received using telemetry (Nexus-D, Medronic, Minneapolis, MN) with a paddle antenna externally positioned over the implanted generator. Packets were programmatically collected using the Nexus-D API for MATLAB.

### 2.3 Signal processing

Raw signals collected from the implanted device were processed using MATLAB (MathWorks, Natick, MA). Data were passed through a 1-Hz high pass 5th order infinite impulse response filter to remove low-frequency drift. The timing of motor-imagery instructions presented to the subject was synchronized with the ECoG data acquisition by marking the signal recording with a low amplitude stimulation pulse delivered to the subject’s scalp. Features for motor imagery classification were extracted from all four channels. For each trial, seven features for each time-series channels 1 and 3 were computed using MATLAB’s *p spectrum* function and averaging power spectral values within predefined frequency bins: 1–8 Hz, 8–12 Hz, 18–26 Hz, 26–35 Hz, 35–45 Hz, 45–70 Hz, and 70–100 Hz (Figure 1B). One feature for each power channel 2 and 4 were computed by averaging the rectified signal output over the course of a trial. Together, these features were used to produce an *M* × *N* (*M* = number of trials, *N* = 16 features were) that was used as input to train a range of distinct classifiers including Random Forests (Breiman, 2001), K-Nearest Neighbors (Cover and Hart, 1967), linear discriminant analysis, a linear support vector machine (Cortes and Vapnik, 1995), and an artificial neural network (Tshitoyan, 2021).

### 2.4 Experimental design

Prior to beginning data collection, the subject underwent tilt table training to allow the subject to acclimate to a standing position and prevent orthostatic hypotension. Across the 10 weeks of study, the study subject participated in 18 sessions. At the beginning of each session, the subject’s blood pressure, heart rate, and pulse oximetry were measured in sitting and standing positions as a baseline to compare with measurements taken between blocks of data collection. Each session was broken up into 1–5 blocks of data collection periods. During each block, the subject performed 10 trials of motor imagery. During each trial, a pair of motor imagery instructions were displayed to the subject on the monitor screen associated with the ReoAmbulator (ReoAmbulator, Motorika, Mount Laurel, NJ; Figure 1C). For each trial, the subject was instructed to think about resting the dominant right hand for about 3 s followed by a motor imagery instruction to think about the moving hand for another 3 s. Following these instructions, the subject would take four to six steps (Figure 1D).

Sessions were divided into open- and closed-loop sessions wherein the first three sessions were open-loop sessions used to collect data and verify our experimental paradigm, and the subsequent 15 sessions were used for closed-loop sessions. Each session consisted of four to five blocks of 10 trials each (Figure 1E). At the beginning of each block of closed-loop decoding sessions, the online classifier was first fit using motor imagery data from the most recently collected two to five blocks, corresponding to around 80 motor imagery observations. The decoder was trained online, meaning it’s parameters were refit after each trial. Thus, for online decoding, training data set was defined as the prior 40–50 trials of data, and the test data set was defined as the live data coming in during the 10 trials during the block. Decoder performance was assessed by calculating the accuracy as shown in Equation 1, where N is the total number of motor imagery instructions, *y_i_* is the motor imagery prompt shown to the subject, and ${\hat{y}}_{i}$ is the predicted value from the model.

**Equation 1**


$$Accuracy=\frac{1}{N}\sum_{i}^{N}1(y_{i}={\hat{y}}_{i})$$

A series of other decoders were trained offline using the same paradigm as the online decoder sessions. For each block of closed-loop data that had been collected, each classifier was trained with the same previous two to five blocks, then tested using the data collected for the current block without being refit for each motor-imagery trial.

### 2.5 Robot-assisted weight supported treadmill training

During closed-loop sessions, decoded values were used to trigger walking of a robot-assisted, body weight-supported treadmill by sending rest and walk commands through a customized microcontroller (ArduinoUno, Arduino, Italy). The robotic treadmill was configured to walk at a speed of 0.6–1.7 km/h for four to six gait cycles before stopping. As the robot was slowing down, another trial would begin issuing a visual cue prompting the subject to think about moving the dominant upper extremity. If a move state was correctly decoded again, the robot would be triggered to resume stepping for an additional four to six gait cycles.

### 2.6 Statistical analysis

Decoder accuracy was defined as the percentage of correctly classified motor imagery states for any set of motor imagery signal data (see Equation 1). Decoding classifiers were trained using 5-fold cross validation. All data were tested for normality using the Shapiro-Wilk Test for normality. Univariate comparisons were computed using the parametric student’s independent T-Test when data was Gaussian, otherwise non-parametric Mann-Whitney U test was performed. Similarly, for assessing variance across conditions, one-way analysis of variance (ANOVA) was used for Gaussian data, and the Kruskal-Wallis test was used as the non-parametric counterpart. The significance level (α) was set to 0.05. When *post hoc* multiple comparisons were performed, Bonferroni correction was applied to the family-wise error rate (α). Correlation between variables was tested using Pearson’s product-moment correlation or Spearman’s rank-order correlation for parametric and non-parametric datasets respectively.

## 3 Results

### 3.1 Cardiovascular monitoring

**Figure 2:**
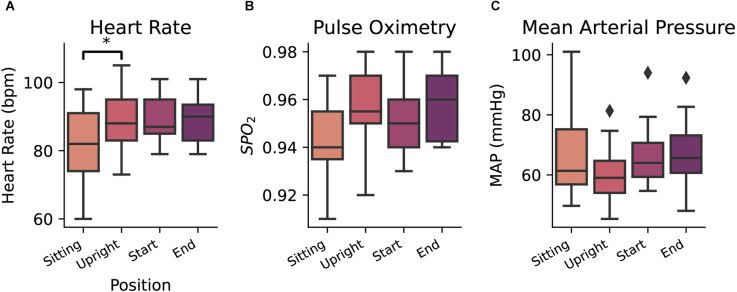
Heart rate **(A)**, pulse oximetry **(B)**, and mean arterial pressure **(C)** measurements taken at four consecutive points (sitting in a wheelchair, upright position in the ambulation harness, at the beginning of the daily session, and at the end of the daily session). Diamond markers indicate data points that lie outside of 1.5 × IQR (Inter quartile range). ^*^p = < 0.05.

The subject’s blood pressure, heart rate, and pulse oximetry, were measured to monitor for adequate orthostatic response to transferring into the standing position and were measured at four time points during each session: (1) at the beginning of each session in the sitting position; (2) once upright in the weight-support harness; (3) immediately before starting the experiment; and (4) sitting at the end of the experiment. Changes in these measurements were relatively stable across these time points. Significant fluctuations with moderate effect size were only noted in heart rate (Figure 2A) between sitting and standing positions (ANOVA *F*_(3,60)_ = 3.47; *p* = 0.02; η^2^ = 0.065, Tukey-HSD p=0.03), indicating that, predictably, as blood pressure drops during standing, the subject’s heart rate on average increased. No differences were found for mean arterial pressure (ANOVA *F*_(3,60)_ = 1.39, *p* = 0.25) and pulse oximetry (ANOVA *F*_(3,57)_ = 2.66, *p* = 0.057;) across the timing of sessions (Figures 2B,C).

### 3.2 BCI system performance

**Figure 3:**
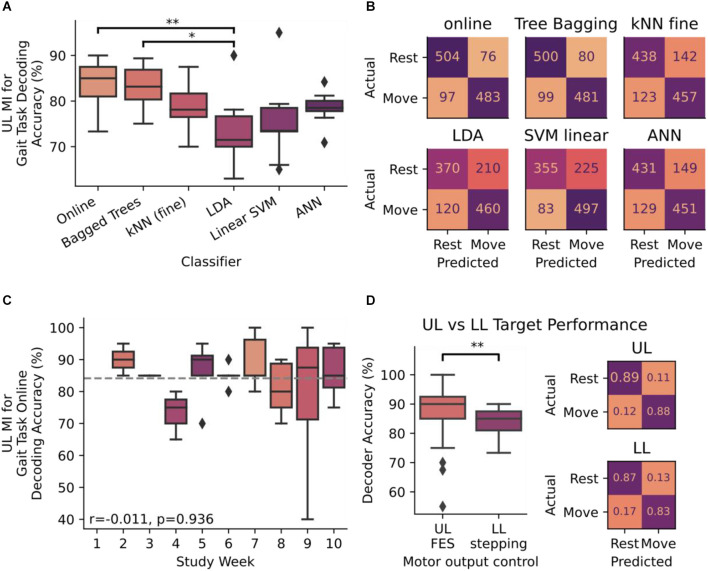
**(A)** Decoder accuracy for decoding upper-limb (UL) motor-imagery (MI) for initiating stepping measured as a ratio of correctly decoded instructions to the total number of instructions presented. Accuracy was measured in six classifiers: online bagged trees, offline bagged trees, Fine K-Nearest Neighbors (kNN), Linear Discriminant Analysis (LDA), Linear Support Vector Machine (SVM), and an Artificial Neural Network (ANN). Differences between each classifier were detected by ANOVA and Tukey-HSD *post hoc* for pairwise analysis. **(B)** Confusion matrices for each of the six classifiers in panel **(A)**. Integer values range from 0 to 580, which is the number of times each motor imagery instruction presented to the subject over the 9 weeks of close-loop analysis in the study. **(C)** Accuracy performance of online bagged trees classifier over the course of the 10 week study period. Gray dotted line indicates the mean value across all weeks (84.15%). The stability of the decoder was measured with accuracy with respect to time and evaluated using Spearman’s correlation coefficient (r). **(D)** Differences in decoder performance, measured by accuracy (%) and confusion matrices. In both instances, bagged-tree classifiers were trained on UL MI and outputs were used for triggering UL FES in the previous study and for triggering gait stepping (LL) in this study (^*^p < 0.05, ^**^p < 0.01). Confusion matrices are normalized to true values as the number of trials completed in this LL assessment differed from the number of trials in the previously performed UL study. Diamond markers in all box plots indicate data points that lie outside of 1.5 × IQR.

Iterative blocks of data were used to train and test an array of motor-imagery classifiers including Random Forests (Breiman, 2001), K-Nearest Neighbors (Cover and Hart, 1967), linear discriminant analysis, a linear support vector machine (Cortes and Vapnik, 1995), and an artificial neural network (Tshitoyan, 2021). These models were trained on data from the extracted features using 5-fold cross validation. Across the 15 closed-loop sessions, 580 trials were performed wherein correctly decoded neural signals were used to trigger walking on the system. The online bagged trees classifier performed the best at 84.15% accuracy on average per week during the study (Figure 3A), better than the standard BCI accuracy criterion of 70% (Kübler et al., 2001; *T* = 8.362, *p* < 0.001). Differences between each classifier were detected using one-way ANOVA (*F*_(5,48)_ = 4.195, *p* = 0.003, η^2^ = 0.304) and Tukey-HSD *post hoc* for pairwise analysis. *Post-hoc* analysis demonstrated that online bagged trees performed significantly better than linear discriminant analysis (Tukey-HSD *p* = 0.0069). Online decoding and bagged trees classifiers were able to distinguish between the MI states consistently, while others had higher error rates when decoding either the rest state alone or both states (Figure 3B). With the online classifier performing best, we assessed the association between time and accuracy and found that performance remained stable across the 9 weeks of close-loop study without the need for retraining (spearman’s *r* = −0.01, *p* = 0.936, Figure 3C).

To determine whether end effector control had a potential role in decoder performance, we compared decoder performance when the subject was targeting LL gait tasks vs. data previously collected while the subject was targeting UL grasping tasks (Cajigas et al., 2021). Decoder performance was significantly different (Mann-Whitney Test: *U* = 797.5, *p* = 0.031) depending on whether MI was directed at UL grasp tasks in contrast to the same signal being used to drive stepping tasks (Figure 3D). In both cases the error rates for predicting each MI state were balanced, meaning the online classifier could accurately detect the movement state similar to the rest state in both experiments.

### 3.3 Cardiovascular measures on BCI performance

**Figure 4:**
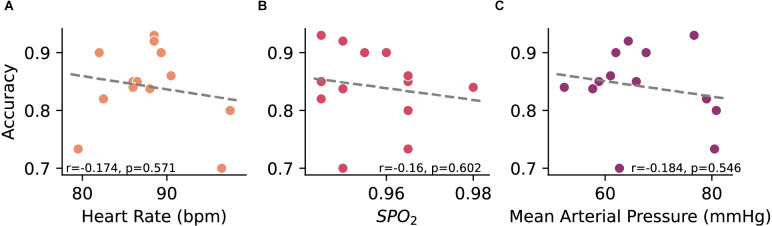
Relationship between online decoder accuracy and measured **(A)** heart rate, **(B)** pulse oximetry, and **(C)** mean arterial pressure. Each point represents a closed-loop data session where the accuracy is an average across all trials and the cardiovascular metrics is the average of pre- and post-session measurements. Linear regression between the variables was performed using Pearson’s product-moment correlation.

Cardiovascular dynamics, particularly blood pressure, can affect the cortical activity and arousal (Duschek and Schandry, 2007), potentially affecting the ability to accurately decode motor-imagery states from EEG. Given the variability in cardiovascular dynamics during sessions, we examined associations between decoder accuracy and cardiac measurements. Heart rate, pulse oximetry, and mean arterial pressure metrics taken at the beginning of each session (*n* = 18) were plotted against the average decoding accuracy for a given session to determine whether cardiovascular changes are associated with decoder performance. A nominal negative association was found between accuracy and heart rate (*r* = −0.174), pulse oximetry (*r* = −0.16), and mean arterial pressure (*r* = −0.184) though none of these were statistically significant (Figure 4).

Additionally, informal discussion with the subject indicated that the subject seldomly became fatigued and that most difficulties arose from physical changes in posture, and that completing motor-imagery tasks were easily performed.

## 4 Discussion

In this study, we demonstrated the ability of a subject with cervical SCI to accurately control the initiation of stepping within a robotic exoskeleton. With adequate prior tilt-table training, the subject was able to tolerate an upright posture and use the currently implanted BCI system aimed at restoring upper extremity control to also control stepping within a robotic treadmill system. Much of the prior work in the BCI literature focuses on upper-extremity control (Robinson et al., 2021), thus implementing models of LL-driven BCI applications is needed to elucidate current technological, methodological, and scientific limitations of BCI in aiding movement reanimation and restoration after paralysis. Additionally, among LL BCI work, almost all studies have used non-invasive EEG (He et al., 2018) and here we accomplished motor imagery decoding through fully implanted ECoG electrodes. Importantly, as individuals with SCI are potential users of BCI technology (Anderson, 2004; Collinger et al., 2013), enabling LL control could benefit all patients with sustained SCI by expanding the use of upper extremity motor-imagery to enable control of lower extremity function.

With the electrodes implanted over the sensorimotor hand region, the participant imagined motor movements of their upper extremity to induce stepping of the lower extremity. This demonstrates that the control signal used for UL movement can be accurately decoded to initiate the LL control mechanism, demonstrating versatile multi-functional use of the same signal that could be used to engage in upper-extremity motor control–an important characteristic that BCI users desire (Huggins et al., 2015).

A unique component of BCI applications of ambulatory exoskeletons and treadmills is the potential effects of cardiovascular autonomic dysregulation on system performance and patient tolerance. Understanding physiological changes is important for LL control where patients’ cardiovascular regulation is perturbed by transferring from a seated to a standing position. In addition to orthostatic changes, simply engaging in motor imagery activity has shown to have cardiovascular effects (Collet et al., 2013; Peixoto Pinto et al., 2017; Lanata et al., 2020), but whether this effect and its influence on decoding performance persists in SCI patients, especially in upright positions, is not fully elucidated. Despite this, this study demonstrates that decoding is feasible and stable for SCI patients in this setting, though more work is warranted to determine the extent to which cardiovascular dynamics, body position, and end effector motor imagery mismatch might affect general neural signal decoding.

### 4.1 Limitations

Workshops within the BCI research community have identified several performance measures for evaluating BCI technology (Huggins et al., 2014; Thompson et al., 2014): accuracy, including information transfer rate, decoding timing, and latency. Though the participant successfully, and accurately, triggered the gait cycles of the weight-supported treadmill, engineering constraints on the ReoAmbulator limited the ability to measure timing delays in the system; e.g., time to decode and trigger the robotic exoskeleton. In this study, however, our objective was to confirm the accurate decoding of motor-imagery for gait control. Yet, as research in continuous control of exoskeletons and assistive walking devices moves forward, interpreting timing effects for translational use will be important to determine and ensure continuous performance in long-term studies.

The clinical efficacy of BCI rehabilitation paradigms relies on a close relationship between robot-controlled movement and movement intention (Robinson et al., 2021). The subject in this work utilized motor imagery of the UL to drive control of LL movements. Though useful from an assistive device perspective, the mismatch of motor imagery and assisted motor output becomes important from a rehabilitation perspective considering the visual, proprioceptive, and sensorimotor feedback effects on both rehabilitation outcomes and BCI performance when temporally coupled to the intended movement (Ramos-Murguialday et al., 2012; Frost et al., 2015). Through this method of temporal coupling, BCI research has shown promise to induce adaptive plasticity for recovery (Ethier et al., 2015; Jovanovic et al., 2021). The mixed paradigm in this study, however, makes elucidating the effects of UL vs LL rehabilitation difficult. It will be important to design studies to understand whether this mixed paradigm for upper and lower extremity rehabilitation is effective across patients.

Only one subject participated in this study to demonstrate reliable decoding of lower-limb control from motor imagery. Signal decoding relied on event-related desynchronization (ERD) and other spectral features, a phenomenon that is well studied in both subjects with and without paralysis in UL (Pfurtscheller, 1997; López-Larraz et al., 2015; Gant et al., 2018) and LL control (Donati et al., 2016; Shokur et al., 2018). Ultimately, whether accurate, prolonged decoding while standing in SCI subjects can generalize to the broader SCI population remains an important study topic for BCI research targeting LL control. Yet, preliminary work from our group demonstrated that ERDs were observed in individuals after SCI and that this could be used as a reliable trigger when observed *via* EEG (Gant et al., 2018). Therefore, the likelihood that such a methodology can be used across individuals is likely.

Our study paradigm obtained MI signals from the subject by presenting instructions to the subject in a fixed pattern (rest then move) and at fixed time intervals (3 s). Subsequent work in our group has established real-time decoding for UL control (Cajigas et al., 2021). This real-time decoding can detect MI without the need to cue the subject to begin performing MI by recognizing the temporal patterns of specific MI states within the signal as it is sampled. Future work will need to investigate real-time decoding algorithms for BCIs targeting LL control. Such real-time decoding for LL control will be necessary to enable subjects to volitionally control ambulatory devices at will and for longer durations beyond the time confined to cue presentation.

## 5 Conclusion

BCIs may prove to be valuable assistive devices for motor impaired individuals. Using four channels of ECoG signals from a fully implanted BCI system enabled a patient with cervical SCI to trigger walking on a weight-supported treadmill, and the decoder’s performance persisted throughout the duration of the study. Work is still needed to more rigorously evaluate the long-term effects of gait-based BCI applications and to determine their clinical implications as assistive devices, and in rehabilitation protocols.

## Data Availability Statement

The raw data supporting the conclusions of this article will be made available by the authors, without undue reservation.

## Ethics Statement

The studies involving human participants were reviewed and approved by University of Miami Institutional Review Board. The patients/participants provided their written informed consent to participate in this study.

## Author Contributions

IC, NP, JJ, and AP developed the experimental design of the study. IC, NP, JN, and SG developed the system to link the implant to drive the ReoAmbulator and collected data. LF coordinated subject involvement in the associated clinical trial. MI and JJ performed prior surgical implantation and patient care. KD wrote the manuscript. IC, NP, and KD analyzed the data. All authors contributed to the article and approved the submitted version.
